# The Evolving Role of Esmolol in Management of Pre-Hospital Refractory Ventricular Fibrillation; a Scoping Review

**Published:** 2020-02-25

**Authors:** Dennis Miraglia, Lourdes A. Miguel, Wilfredo Alonso

**Affiliations:** 1 Department of Internal Medicine, Good Samaritan Hospital, Aguadilla, PR, United States

**Keywords:** Cardiopulmonary resuscitation, esmolol, out-of-hospital cardiac arrest, ventricular fibrillation

## Abstract

**Introduction:**

Few studies have described their experience using esmolol, an ultra-short acting β-adrenergic antagonist, in the emergency department (ED) as a feasible adjuvant therapy for the treatment of refractory ventricular fibrillation/pulseless ventricular tachycardia (VF/pVT) out-of-hospital cardiac arrest. However, there is currently insufficient evidence to support the widespread implementation of this therapy. The aim of this scoping review was to summarize the current available evidence on the use of esmolol as an adjuvant therapy for refractory VF/pVT out-of-hospital cardiac arrest, as well as to identify gaps within the literature that may require further research.

**Methods:**

We conducted a comprehensive literature search of MEDLINE via PubMed, Embase, Scopus, and the Cochrane Central Register of Controlled Trials (CENTRAL) on July 5, 2019. The search was restricted to articles that were published from January 2000 to July 2019. Google Scholar was searched and reference lists of relevant papers were examined to identify additional studies. We included any controlled clinical study design (randomized controlled trials and non-randomized controlled trials) and observational studies (cohort studies and case-control studies) in adults providing information on the use of esmolol as an adjuvant therapy for refractory VF/pVT out-of-hospital cardiac arrest.

**Results:**

The search yielded 2817 unique records, out of which 2 peer-reviewed articles were found relating to the research purpose totaling 66 patients 33.3% (n = 22) of which received esmolol. These studies found that sustained return of spontaneous circulation (ROSC) was significantly more common in the patients that received esmolol compared to the control group. However, no statistically significant outcomes were found regarding survival to discharge and favorable neurological outcome. No randomized controlled trials were identified.

**Conclusion:**

To date, it is difficult to conclude the real benefit of esmolol as an adjuvant therapy for refractory VF/pVT out-of-hospital cardiac arrest based on the available evidence. The findings of this scoping review suggest that there is a paucity of research and limited evidence to support this therapy.

## Introduction

In 2016, the annual estimated incidence of emergency medical services (EMS)-assessed out-of-hospital cardiac arrest reported by the Resuscitation Outcomes Consortium (ROC) Epistry for cardiac arrest was about 356,500 people of all ages in the United States (US). Among EMS-treated out-of-hospital cardiac arrest patients, about 21% had shockable rhythms of ventricular fibrillation/pulseless ventricular tachycardia (VF/pVT). Survival to hospital discharge after EMS-treated cardiac arrest was about 11.4%, while this rate was 37.4% for bystander-witnessed VF cardiac arrest in patients of all ages (1, 2).

VF is a life-threatening arrhythmia that could lead to sudden cardiac death if not treated emergently. Currently, the American Heart Association (AHA) recommends immediate electrical defibrillation as the most effective treatment for VF/pVT (3, 4). However, there is a subgroup of patients in which VF remains refractory to standard electrical defibrillation (5, 6). Refractory VF is defined as VF unresponsive to at least three standard defibrillation attempts and Advanced Cardiovascular Life Support (ACLS); however, currently, neither a clear consensus to the definition of refractory VF nor best-established practices for the management of refractory VF exist (7). Since there is no consistent definition for refractory VF in the clinical literature, we used the one cited above throughout this review.

Few observational studies have described their experience with pre-hospital double defibrillation to treat refractory VF/pVT. These studies have been unable to reach a conventional threshold for statistical or clinical significance, although this does not mean that it is safe to conclude that there is no difference (8-11). There may be better strategies for treating pre-hospital refractory VF/pVT than the use of double defibrillation. Incorporation of mechanical chest compressions devices (LUCAS) and earlier deployment of extracorporeal membrane oxygenation (ECMO) assisted revascularization have shown promising results. Therefore, ECMO has been increasingly used as a bridge to definitive treatment including revascularization (CAG and PCI) in patients with refractory cardiac arrest (12-20). The beta-blocker, esmolol has been recently studied as a feasible adjuvant therapy for patients during both electrical storm (ES) and refractory VF/pVT; offering a potential lifesaving treatment option for these patients (21-26). However, the limited evidence about the rationale behind the use of intravenous esmolol for the treatment of refractory cardiac arrest makes it a unique and unproven therapy still to be proven.

Consequently, there seems to be a need to review the literature regarding the use of esmolol for refractory VF/pVT out-of-hospital cardiac arrest in more detail to: identify areas for future research, and to develop strategies for a clinical protocol that will offer a potential lifesaving treatment option for this specific patient population, which is in accordance with the recommendations in the current clinical guidelines. A scoping review was deemed most appropriate because it is exploratory in nature. All methodologies will be considered in the process of this review, as there are few studies evaluating esmolol for refractory VF/pVT out-of-hospital cardiac arrest. Therefore, the scoping review will identify the feasibility of future work in this area from a variety of methodological perspectives. This paper will summarize the state of the current literature on the use of esmolol in the emergency department (ED) as a feasible adjuvant therapy for refractory VF/pVT out-of-hospital cardiac arrest and identify gaps that will provide direction for future research in the area.

## Methods

We followed the PRISMA-ScR (Preferred Reporting Items for Systematic reviews and Meta-Analyses extension for Scoping Reviews) guidelines developed following published guidance from the EQUATOR Network (Enhancing the QUAlity and Transparency Of health Research) (27) and the methodological framework for conducting a scoping review developed by Arksey and O’Malley (28). A scoping review protocol was not drafted. A scoping review is the process of mapping the main concepts of a research area to its source and evidence available in the literature. The five stages the authors developed were followed in order to maintain a transparent method for data collection, analysis, and interpretation: 1) identifying the research question; 2) identifying relevant studies; 3) selecting studies; 4) charting the data; and 5) collating, summarizing, and reporting the results (28).

***Stage 1: Identifying the Research Question***

The aim of this scoping review was to gain a clear understanding of the current available literature on the use of esmolol in the ED as an adjuvant therapy for patients with refractory VF/pVT out-of-hospital cardiac arrest. A scoping review should be undertaken to determine the value of undertaking a full systematic review and forms part of the complex intervention framework (29, 30). The research objectives of this review were to: 1) summarize the current base of evidence on this intervention for refractory VF/pVT out-of-hospital cardiac arrest; 2) identify gaps in the literature that may require further research. The review questions for this scoping review were formulated following the PICOT method. P (Population) - people (≥18 years old) who suffer from refractory VF/pVT out-of-hospital cardiac arrest, I (Intervention) - a pharmacology intervention (esmolol), C (Comparator) - no esmolol (control), O (Outcomes) - survival to discharge and favorable neurological outcome and long-term survival and favorable neurological outcome, T (Time) - all studies from January 2000 to July 2019 were considered. Studies were not limited according to the time of follow-up.

***Stage 2: Identifying Relevant Studies***

***Databases***

We searched the following databases for eligible articles on July 5, 2019: MEDLINE via PubMed, Embase, Scopus, and the Cochrane Central Register of Controlled Trials (CENTRAL), as well as the reference lists of all selected articles. Additionally, Google Scholar was searched for any additional citations. Finally, we searched for unpublished or ongoing clinical trials using the WHO International Clinical Trials Registry (WHO ICTRP), and the ClinicalTrials.gov registry on July 12, 2019. The search was repeated one month prior to submission for publication to ascertain that no new literature was published in the interim. We believed these four search databases would reach all the relevant journals within the area of interest. Overall, 2817 articles were found using the search terms and databases.

***Search Terms***

The search strategy was developed by two investigators (DM and LM), with the help of healthcare librarians. We used the PRESS (Peer Review of Electronic Search Strategies) checklist to develop the research strategy (31). The search strategy was developed according to keywords related to esmolol in combination with “out-of-hospital cardiac arrest” and “refractory ventricular fibrillation”. Keywords used in the search were based on the implemented PICO model, which was first defined for use in MEDLINE via PubMed and subsequently adapted for the other databases. The example of PubMed research query was: (((“cardiopulmonary resuscitation”[Title/Abstract] OR “CPR”[Title/Abstract] OR “management”[Title/Abstract] OR “treatment”[Title/Abstract] OR “pre-hospital cardiac arrest”[Title/Abstract] OR “out-of-hospital cardiac arrest”[Title/Abstract] OR “emergency department”[Title/Abstract] OR “ED”[Title/Abstract])) AND ((“sudden”[Title/Abstract] AND “death”)[Title/Abstract] OR “refractory ventricular tachycardia”[Title/Abstract] OR “Refractory ventricular fibrillation”[Title/Abstract] OR “RVT”[Title/Abstract] OR “RVF”[Title/Abstract] OR “pulseless ventricular tachycardia”[Title/Abstract] OR “pVT”[Title/Abstract] OR “ventricular fibrillation”[Title/Abstract] OR “ventricular arrhythmia”[Title/Abstract] OR “heart arrest”[Title/Abstract] OR “cardiac arrest”[Title/Abstract])) AND “esmolol”[Title/Abstract] OR “beta-blockade”[Title/Abstract].

***Stage 3: Study Selection***

***Inclusion and Exclusion Criteria***

We used the PCC (Population, Concept, and Context) framework to delineate eligibility criteria (32). Studies were eligible for inclusion if they reported on the use of esmolol as an adjuvant therapy for patients (≥18 years old) undergoing resuscitation for refractory VF/pVT out-of-hospital cardiac arrest and included any controlled clinical study design (randomized controlled trials and non-randomized controlled trials) and observational studies (cohort studies and case-control studies) with a control group (i.e. patients not receiving esmolol) and were published between January 2000 and July 2019. This time period was selected because a preliminary review suggested there would not be any relevant articles prior to the year 2000. Studies were excluded if they were not written in English, included in-hospital cardiac arrest, reported on animal studies, reported on traumatic cardiac arrest, reported on pediatric cardiac arrest, reported cardiac arrests in pregnancy, and patients had received esmolol for arrhythmias other than VF/pVT.

**Figure 1:**
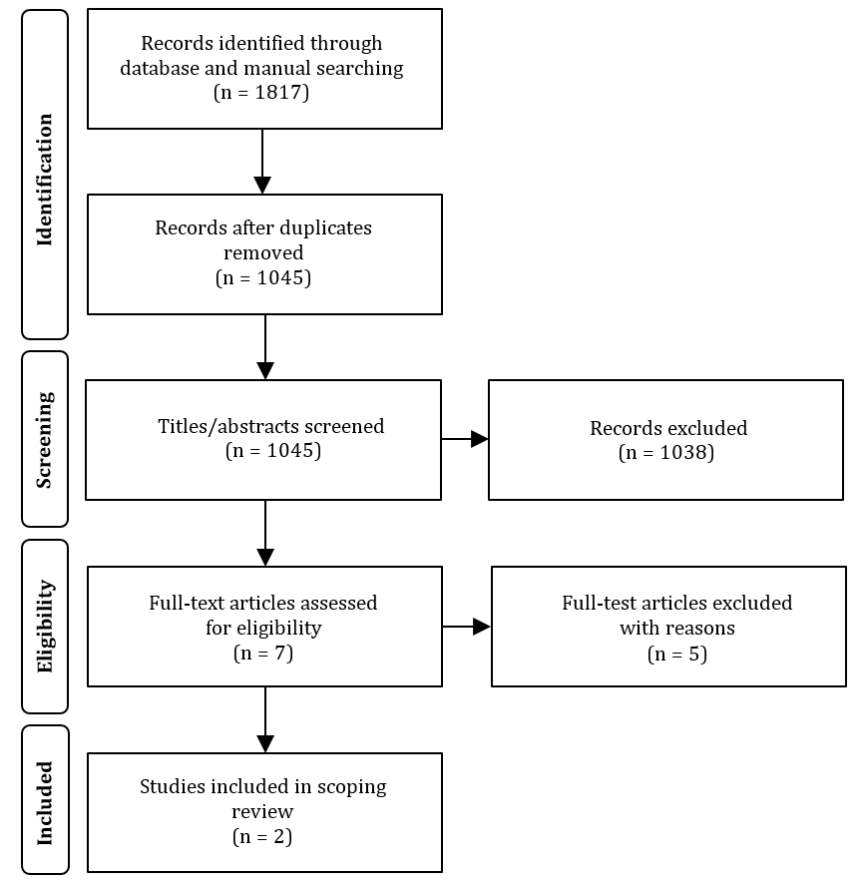
Preferred Reporting Items for Systematic Reviews and Meta-Analyses flowchart

The databases were searched by one author (DM). Following the search and the automatic removal of duplicates, the titles and abstracts were subsequently appraised for eligibility by two independent authors (DM and LM). The full texts of titles and abstracts were reviewed for studies that were considered potentially relevant. Any discrepancies regarding the selection of articles retained for full-text review were resolved by discussion with a third reviewer (WA). The selection process is described in Figure 1.

***Stage 4: Charting the Data***

The charting of data is a descriptive-analytical method that is used to extract the information from individual articles (28). Excel 2019 (Microsoft, Redmond, WA) was utilized for this stage. We collected descriptive characteristics such as first author, year of publication, study period, the country where the study was held, study design, research setting, study population, sample size, measures, interventions, key findings, and limitations. Data for all reported outcomes were extracted from every study included in the review by two independent reviewers (DM and LM). Discrepancies regarding the extracted data were resolved by discussion with a third reviewer (WA). Descriptive statistics were summarized by presenting the median (IQR) for continuous variables and number and percentage for categorical variables. Table 1 provides an overview of the articles selected for inclusion.

***Stage 5: Collating, Summarizing, and Reporting the Results***

A total of 7 studies were identified as relevant to the review. Two studies were noted to be directly related to the use of esmolol as an adjuvant therapy for refractory VF/pVT out-of-hospital cardiac arrest. The remaining 5 studies were applicable to in-hospital cardiac arrest. These studies were excluded at this level because different types of beta-blockers other than esmolol were used as adjuvant therapy. The discarded articles were approved by the authors before the qualitative analysis was completed. Finally, a total of 2 observational studies were included. Tables 2–4 summarize details of the studies according to demographics, presentation, resuscitative parameters, and outcomes.

***Ongoing Consultation***

It is suggested that a scoping review should include the consultation of experts in the area of research (28). Consultation was not included in this study due to this approach being relatively new and esmolol being used off-label with no information available from randomized controlled trials.

## Results

***Study Populations and Settings***

The initial electronic database search yielded 2817 records. We first removed 1274 duplicates and then eliminated 1536 papers following inspection of the titles and abstracts. We read the full text of each of the 7 remaining articles. Following the inclusion criteria outlined above, 2 retrospective observational studies were found relating to the research purpose totaling 66 patients, 33.3% (*n* = 22) of which received esmolol (24, 25). No randomized or non-randomized controlled trials on esmolol for refractory VF/pVT out-of-hospital cardiac arrest were identified or ongoing at the time of the search.

All studies were published between 2014 and 2016, while patient enrollment periods extended to as early as 2011. Both studies were conducted in a single center with percutaneous coronary intervention capability, but at different institutions. One study was performed in the US (24) and 1 in South Korea (25). Regarding demographics, all articles identified patients as having pre-hospital or ED refractory VF/pVT at some point during the cardiac arrest. The sample sizes of the patients that received esmolol ranged from 6 to 16, the median age of the patients ranged from 54 to 58 years, and the percentage of males ranged from 87.5% to 100%. Most cardiac arrests were witnessed by a bystander, who initiated cardiopulmonary resuscitation (CPR) and were attended by EMS providers.

***Focus and Outcomes***

There was no substantial heterogeneity of outcome measurement across studies. The definition of refractory VF and a clear protocol was uniform across studies. All studies compared the use of esmolol (intervention) with no esmolol (control) in adult patients with refractory VF/pVT out-of-hospital cardiac arrest. Both studies enrolled patients with pre-hospital cardiac arrest and diagnosis of VF/pVT who did not respond to at least 3 deﬁbrillation attempts, 3 mg of epinephrine, 300 mg of amiodarone and remained in arrest upon ED arrival. All patients in the esmolol group received a loading dose of 500 mcg/kg esmolol, followed by a continuous infusion of 0–100 mcg/kg/min (24, 25).

All studies reported on sustained return of spontaneous circulation (ROSC >20 min of spontaneous circulation without recurrence of cardiac arrest) (33), total ED CPR time, total CPR time, survival to intensive care unit (ICU) admission, survival to discharge and favorable neurological outcome. One study reported data about the predominance of acute coronary syndrome and emergency coronary revascularization (24). One study reported on survival and favorable neurological outcomes 30 days, 3 months, and 6 months later (25). Overall, both studies found that sustained ROSC was significantly more common in patients that received esmolol compared to the control group. However, no statistically significant outcomes were found regarding survival to discharge and favorable neurological outcome. Neurological outcomes were evaluated using the Glasgow-Pittsburgh cerebral performance category (CPC) scale. Good neurological outcomes were defined as a CPC score of 1–2 (34, 35).

The first study was published in 2014 by driver et al. Overall, in this study 5 (83.8%) of the patients in the esmolol group had witnessed arrest and 5 (83.8%) had VF as the first documented heart rhythm before receiving esmolol. Furthermore, VF was successfully terminated into sustained ROSC in 4 (66.7%) patients. In addition, 83.8% received emergent cardiac catheterization and 60% of them were diagnosed as having a ST segment elevation myocardial infarction (STEMI). Ultimately, 3 (50%) of the patients included in this study had a CPC score equal to 1 or 2 on discharge. The authors reported that sustained ROSC was significantly more common in the esmolol group compared to the control group (66.7% vs. 31.6%). However, there were no significant differences in survival rates (50% vs. 15.8%), and good neurological outcomes between the two groups (50% vs. 10.5%). All Patients in this study received conventional CPR by ﬁrst responders and most had automated CPR with a LUCAS device (Physio-control) and an impedance threshold device (ITD) (ResQPOD^TM^) when they were transported to the ED (24).

The second study was published in 2016 by Lee et al. Overall, in this study 14 (87.5%) of the patients in the esmolol group had witnessed arrest and 11 (68.8%) had VF as the first documented heart rhythm before receiving esmolol. Furthermore, VF was successfully terminated into sustained ROSC in 9 (56.3%) patients. In addition, 93.8% of the arrests had cardiac origin, but the study did not report if any of the patients suffered from acute coronary syndrome or received emergent reperfusion therapy. Ultimately, 3 (18.8%) of the patients included in this study had a CPC score equal to 1 or 2 on discharge, and 30 days, 3 months and 6 months later. This study found similar results to the study described above. The authors reported that sustained ROSC was significantly more common in the esmolol group compared to the control group (56.3% vs. 16.0%, p = 0.007). However, there were no significant differences in survival rates and good neurological outcomes 30 days, 3 months and 6 months later (18.8% vs. 8%, p = 0.36) (25).

**Table 1:** Details of characteristics and outcomes of studies included in the scoping review

**Author, year** **Country**	**Design, setting, and participants**	**Intervention**	**Key Results**
Driver et al. 2014 United States of America (24)	A retrospective investigation from January 2011 to January 2014 in an urban academic ED. This study included 25 patients (≥18 years old) with out-of-hospital or ED cardiac arrest with refractory VF/pVT who were resistant to at least ≥3 defibrillations, 3 mg of epinephrine, and 300 mg of amiodarone. Six patients received esmolol (intervention) in the ED during CA and were compared to those who did not (control).	Patients received esmolol 500 mcg/kg bolus followed by a 0–100 mcg/kg/min maintenance infusion.	Key outcomes of patients who received esmolol (n = 6) compared with those who did not (n = 19). The esmolol group exhibited better rates of temporary ROSC and survival to ICU admission. When comparing survival rates and survival with favorable neurological outcome, the patients that received esmolol had better outcomes than those who did not. However, no statistically significant outcomes were found in survival to discharge and favorable neurological outcome. Overall, 4 (66.7%) in the esmolol group vs. 6 (31.6%) in the control group had sustained ROSC and survived to ICU admission, respectively. Three (50%) vs. 3 (15.8%) survived to hospital discharge and 3 (50%) vs. 2 (10.5%) survived to discharge with a CPC ≤ 2.
Lee et al. 2016 South Korea (25)	A retrospective single-center pre-post study that evaluated records from January 2012 to December 2015. This study included 41 patients (≥18 years old) with refractory VF out-of-hospital cardia arrest who were resistant to ≥3 defibrillations, 3 mg of epinephrine, 300 mg of amiodarone, and had no ROSC after >10 min of CPR). Sixteen patients received esmolol (intervention) at the ED during CA and were compared to those who did not (control).	Patients received esmolol 500 mcg/kg bolus followed by a 0–100 mcg/kg/min maintenance infusion.	Key outcomes of patients who received esmolol (n = 16) compared with those who did not (n = 25). Sustained ROSC was significantly more common in the esmolol group, compared to the control group (p = 0.007). The esmolol group also exhibited better rates of temporary ROSC and survival to ICU admission. However, there were no significant differences in the rates of survival to discharge and favorable neurological outcome (p = 0.36). Overall, 9 (56.3%) in the esmolol group vs. 4 (16%) in the control group had sustained ROSC and survived to ICU admission, respectively. Three (18.8%) vs. 2 (8%) survived to discharge and had a CPC ≤ 2 at 30, 90, and 180 days.

CA = cardiac arrest; CPC = cerebral performance category; ED = emergency department; ICU = intensive care unit; pVT = pulseless ventricular tachycardia; ROSC = return of spontaneous circulation; VF = ventricular fibrillation; CPR = cardiopulmonary resuscitation.

Notes: Neurological outcomes were evaluated using the Glasgow-Pittsburgh cerebral performance category (CPC) scale. Favorable neurological outcomes were defined as a CPC score of 1–2.

**Table 2:** Esmolol in out-of-hospital cardiac arrest patients with refractory VF/pVT

**Author, ** **Year** **Country**	**Study design**	**Patients,** **n**	**Received** **esmolol,** **n**	**Age,** **median** **(IQR), yr**	**Male,** **n (%)**	**Witnessed arrest** **n (%)**	**Bystander CPR,** **n (%)**	**Initial rhythm VF,** **n (%)**	**SD attempts,** **median** **(IQR)**
Driver et al. 2014 USA (24)	RO	25	6	54.5 (47–59)	6 (100)	5 (83.3)	3/4^a b^	5 (83.3)	6.5^c^ (5–9.5)
Lee et al. 2016 SK (25)	RO	41	16	58 (45.8–72)	14 (87.5)	14 (87.5)	11 (68.8)	14 (87.5)	6 (6–8.75)

CPR = cardiopulmonary resuscitation; RO = retrospective observational; SD = standard defibrillation; VF = ventricular fibrillation; VT = ventricular tachycardia; USA = United States of America; SK = South Korea.

Notes: Total percentages refer to studies with available data. All continuous variables are reported as median interquartile range (IQR) unless specified otherwise

^a ^Refers to the patient who arrested in the ED; one patient was awake on EMS arrival, then arrested.

^b ^Refers to mechanical CPR with LUCAS device.

^c ^Refers to implantable cardioverter-defibrillator (ICD) ﬁrings (approximately every 2-3 min) until it failed 30 min after ED arrival; does not include ICD firings for one patient.

**Table 3:** Esmolol in the out-of-hospital cardiac arrest patients with refractory VF/pVT

**Author, year** **Country**	**Adrenaline, (mg), median (IQR)**	**Amiodaron, (mg), median (IQR)**	**Esmolol ** **loading dose (mcg/kg)**	**Esmolol ** **Drip, (mcg/kg/min)**	**Total CPR time (min), median (IQR)**	**Temporary ROSC** ^ a^ **, ** **n (%)**	**Sustained ** **ROSC** ^ b^ **, ** **n (%)**	**Survival to ICU admission,** ** n (%)**
Driver et al. 2014 USA (24)	6 (5–7.75)	375 (300–450)	500	0–100	63 (57–83)	4 (66.7)	4 (66.7)	4 (66.7)
Lee et al. 2016 SK (25)	6 (3.3–9)	450 (300–450)	500	0–100	55 (35.3–70.3)	13 (81.3)	9 (56.3)	9 (56.3)

CPR = cardiopulmonary resuscitation; ICU = intensive care unit; ROSC = return of spontaneous circulation; VF = ventricular fibrillation; VT = ventricular tachycardia; USA = United States of America; SK = South Korea.

Notes: Total percentages refer to studies with available data. All continuous variables are reported as median interquartile range (IQR) unless specified otherwise.

^a^ Refers to non-fleeting spontaneous circulation lasting >30 seconds but <20 minutes.

^b^ Refers to 20 min of spontaneous circulation without cardiac arrest.

**Table 4:** Esmolol in the out-of-hospital cardiac arrest patients with refractory VF/pVT

**Author, ** **year** **Country**	**Survival to** **discharge,** **n (%)**	**30-day ** **Survival,** **n (%)**	**3 and 6 months Survival,** **n (%)**	**CPC ≤ 2 at** **discharge** **,** **n (%)**	**CPC ≤ 2 at 30** **days** **,** **n (%)**	**CPC ≤ 2** ** at 3 and 6** **months,** **n (%)**
Driver et al. 2014 USA (24)	3 (50)	…	…	3 (50)	…	…
Lee et al. 2016 SK (25)	3 (18.8)	3 (18.8)	3 (18.8)	3 (18.8)	3 (18.8)	3 (18.8)

CPC = cerebral performance category; ellipses (...) = data not available; VF = ventricular fibrillation; VT = ventricular tachycardia; USA = United States of America; SK = South Korea.

Notes: Total percentages refer to studies with available data.

## Discussion

This scoping review sought to describe the available evidence and identify the gaps in the literature on the use of esmolol as a feasible adjuvant therapy for the treatment of refractory VF/pVT out-of-hospital cardiac arrest. Our scoping review revealed that only 2 studies have evaluated the use of esmolol as an adjuvant treatment for adult patients with refractory VF/pVT out-of-hospital cardiac arrest. Both of these studies showed that sustained ROSC was significantly more common in the esmolol group, compared to the control group. Survival to discharge and favorable neurological outcome were at least 2-fold better in the esmolol group, compared to the control group, although these increases were not statistically significant. One study did not report data regarding 30-day, 6-month and long-term survival and favorable neurological outcome (24). The findings of these studies suggest that esmolol may considerably improve the probability of successful ROSC, but not survival, as approximately 2/3 of the patients that had sustained ROSC did not survive to discharge. 

A major limitation discovered in this review is the paucity of research and lack of literature to support this therapy. We identified these as the main important gaps in the available literature. Furthermore, our review revealed that all studies were observational in nature and conducted on a small number of patients. In addition, we did not identify any studies assessing the cost-effectiveness of esmolol for pre-hospital refractory VF. We did not identify any registered or ongoing clinical trials on esmolol for refractory VF out-of-hospital cardiac arrest on the International Clinical Trials Registry Platform. Despite these limitations, the findings from this review highlight an area of research that may contribute to improving survival of people with refractory VF/pVT out-of-hospital cardiac arrest, but would need to be investigated in a more robust manner.

Epinephrine has been a longstanding treatment for cardiac arrest patients; yet, the literature has not shown an increase in survival rates when it has been used in higher doses (36). During prolonged resuscitation from cardiac arrest there is an increase in sympathetic tone, at least partially, due to the mechanism of epinephrine. The activation of ß-adrenoreceptors by epinephrine causes up to 4-fold increase in myocardial oxygen consumption in patients with VF/pVT via its positive chronotropic and inotropic effects (37, 38). In addition, coronary blood flow may be reduced to up to 40%, increasing myocardial ischemia (37).

Esmolol has shown promising results to support the effectiveness of beta-1 selective blockade in refractory VF/pVT (21, 23-26). Esmolol as an adjuvant therapy may be an alternative treatment for these patients since it is an ultra-short acting beta-1 selective adrenergic receptor blocker and a perfect sympatholytic agent, which is extremely cardioselective and has a quick onset of action. It has the fastest onset (90 seconds) and the shortest half-life ([t1⁄2] = 9 minutes) among beta-blockers (39). Esmolol is also able to mitigate the depression of VF threshold produced by high doses of epinephrine used during cardiac arrest, due to its ability to dampen the sympathetic tone, which is one of the proposed mechanisms behind the use of esmolol for refractory VF. Due to its quick onset and offset, it is ideal for these patients, without having the excessive/prolonged effects of the drug during and after resuscitation (37-40). When esmolol is administered as a bolus, it is followed by a continuous infusion, the onset of activity occurs within 2 minutes, with 90% of b-blockade at 5 minutes (39). Generally, for cardiac arrest patients, a loading dose of 500 mcg/kg over one minute has been administered prior to a maintenance infusion dose of 0–100 mg/kg/min (24, 25). 

Esmolol, as an adjuvant therapy for refractory VF/pVT, could be easily used in the ED and in-patient hospital settings. However, esmolol is not readily available for pre-hospital use, and, as a result, patients who experience refractory out-of-hospital cardiac arrest are reliant on rapid transportation to the closest hospitals prepared to handle these types of patients. Hence, despite the current advances in pre-hospital care and the feasibility to provide quick access to perfusion/reperfusion therapies, the main goal of pre-hospital care for patients with refractory cardiac arrest is rapid transport to definitive care while supporting patients (18-20, 41). The results of these studies are not to be generalized as these studies were performed at a single medical center and had a small sample size; however, these studies showed a signal of benefit and a feasible adjuvant treatment strategy for one of the most difficult challenges of resuscitative medicine.

## Limitations

This study had some limitations. First, the primary limitation of the scoping review methodology is the lack of quality assessment of the included articles. However, the goal of a scoping review is simply to identify research that has been conducted, not necessarily to assess quality (28). While the quality assessment was not a goal of the research, quality should be considered before applying these findings in clinical practice. Second, the scientific evidence we used has limitations due to small sample size and the nature of single-centered, retrospective, non-randomized, observational studies with their subjective potential for selection bias. Third, the studies did not report collapse time to esmolol bolus or maintenance infusion; in addition, there was a lack of follow-up in one study and as a consequence, no long-term survival or functional outcomes were reported. Fourth, all of the studies included in this review listed at least two or three limitations in the discussion section of the article, and there is a risk of bias if the authors of the included articles did not mention all the true limitations of their studies. Fifth, there might be considerable differences between EMS and variations in the transport of patients. Finally, the rationale for undertaking an early scoping review is now recognized in that such review has, among other things, the potential to influence the design of future primary studies and systematic reviews. Furthermore, we plan to conduct a systematic review, which will allow us to incorporate studies that have been published after the cut-off date of our searches and thereby, ensure an up to date review on this important topic.

## Conclusion

Current research shows promising results on the use of esmolol as feasible adjuvant therapy for refractory VF/pVT out-of-hospital cardiac arrest. However, there is a paucity of research and a lack of literature to support this therapy. We urgently need studies on esmolol, to identify differences in important clinical outcomes such as survival to discharge and favorable neurological outcome. As studies become available on this topic, they will help us justify its use and application in clinical practice. It is recommended to evaluate these outcomes in randomized controlled trials in order to obtain a higher level of scientific evidence.

## Perspective

As mentioned above, we plan to conduct a systematic review and meta-analysis in a second research to evaluate the efficacy of esmolol in patients with refractory VF/pVT out-of-hospital cardiac arrest.

## Data Availability

All data generated or analyzed during this study are included in this published article.
